# Development and Efficacy Evaluation of Innovative Cosmetic Formulations with *Caryocar brasiliense* Fruit Pulp Oil Encapsulated in Freeze-Dried Liposomes

**DOI:** 10.3390/pharmaceutics16050595

**Published:** 2024-04-27

**Authors:** Letícia Kakuda, Patrícia M. B. G. Maia Campos, Wanderley P. Oliveira

**Affiliations:** Faculty of Pharmaceutical Sciences of Ribeirão Preto, University of São Paulo, Ribeirão Preto 14040-903, Brazil; leticia.kakuda@usp.br (L.K.); pmcampos@usp.br (P.M.B.G.M.C.)

**Keywords:** encapsulation, liposome, *Caryocar brasiliense*, cosmeceuticals, phytotechnology

## Abstract

Encapsulation and drying technologies allow the engineering of innovative raw materials from plant biodiversity, with potential applications in pharmaceutical and cosmetic fields. Lipid-based nanoencapsulation stands out for its efficiency, ease of production, and versatility in encapsulating substances, whether hydrophilic or lipophilic. This work aimed at encapsulating pequi oil in liposomes and freeze-dried liposomes to enhance its stability and functional benefits, such as skin hydration and anti-aging effects, for use in innovative cosmetic formulations. Pequi oil—extracted from the *Caryocar brasiliense* fruit pulp, a plant species from Brazilian plant biodiversity—is rich in secondary metabolites and fatty acids. Liposomes and dried liposomes offer controlled production processes and seamless integration into cosmetic formulations. The physicochemical analysis of the developed liposomes confirmed that the formulations are homogeneous and electrokinetically stable, as evidenced by consistent particle size distribution and zeta potential values, respectively. The gel-type formulations loaded with the dried liposomes exhibit enhanced skin hydration, improved barrier function, and refined microrelief, indicating improvements in skin conditions. These results highlight the potential of dried liposomes containing pequi oil for the development of innovative cosmeceutical products. This research contributes to the valorization of Brazilian biodiversity by presenting an innovative approach to leveraging the dermatological benefits of pequi oil in cosmetic applications.

## 1. Introduction

Brazil, home to 15–20% of the world’s biodiversity, ranks among the 17 mega-diverse countries [1]. Its rich biodiversity spans several biomes, including the Cerrado—a transitional biome renowned for its unique and commercially valuable plant species [2,3]. In the diverse vegetation of Cerrado, pequi oil—extracted from the pulp of *Caryocar brasiliense* Cambess—stands out for its use in traditional medicine and cuisine [2,3]. Pequi oil is rich in fatty acids, like oleic and palmitic acids, antioxidants, and vitamins, possessing potent natural antioxidant activity. It combats oxidative stress, supports cellular homeostasis, and protects the nuclear membrane [4,5]. Moreover, its secondary metabolites, including phenolic compounds, tocopherols, phytosterols, lycopene, and beta-carotene, are known for their antioxidant and anti-inflammatory properties [3,6]. These properties have a key role in combating oxidative stress and promoting skin hydration, leading to improved skin health. In this context, pequi oil, with its rich composition and beneficial properties, emerges as a highly valued ingredient for cosmetic applications. Particularly, it may stand out in products designed to moisturize and preserve the youthful appearance of the skin.

While rich in beneficial compounds, vegetable oils are susceptible to degradation from environmental factors such as air, light, and heat. This degradation can compromise their physicochemical stability, making them unsuitable for consumption or use in the cosmetic and food industries. Excessive heat, in particular, can cause hydrolysis, oxidation, and decomposition of fatty acids, significantly affecting the quality of the oil [7]. In response to these challenges, encapsulation techniques emerge as innovative solutions. They enhance the stability of vegetable oils and protect the bioactive compounds within, ensuring their efficient delivery. This approach maximizes the therapeutic and functional benefits of the oils, making encapsulation an invaluable strategy in extending the shelf-life and efficacy of vegetable oils in various applications [7,8,9]. In particular, the encapsulation of pequi oil can provide a strategic advantage in the cosmetic field by increasing its stability and bioavailability and providing a controlled release. The encapsulation process can also improve the delivery of pequi oil to deeper layers of the skin, enhancing the moisturizing and antioxidant effects of the oil. In this context, the encapsulation of pequi oil ensures its stability, maximizes its potential benefits for the skin, and adds value to the oil and Brazilian biodiversity.

Liposomes, lipid-based systems primarily constituted of phospholipids, are especially noteworthy. These phospholipids spontaneously arrange into bilayers, forming spherical vesicles when dispersed in water [10]. Cholesterol, an essential lipid bilayer component, enhances the vesicle’s fluidity and stability [11]. Encapsulation within liposomes yields numerous benefits for cosmetic applications. It can accommodate hydrophobic and hydrophilic substances, exhibits biocompatibility, and delivers desirable sensory attributes. Due to their structural similarity to cell membranes, liposomes enhance skin hydration and improve the encapsulated substances’ stability and bioavailability [9,12,13].

Incorporating oil into this system alters its apparent aqueous solubility by enclosing the oil within the lipid bilayer formed by the hydrophobic tails of phospholipids. This structure allows for the uniform dispersion of the oil in water, with the hydrophilic heads interacting with the aqueous environment, highlighting the versatile applications of liposome encapsulation in enhancing the functionality of vegetable oils [11,14].

To further enhance the stability and bioactivity of liposomes, removing their water content through processes such as spray drying or freeze drying is a prominent approach, leading to the production of dried liposomes. These particles, once rehydrated, reassemble into liposomes, offering advantages like increased physical stability and extended shelf life without compromising their intrinsic properties [15]. This process facilitates controlled and scalable production and simplifies the integration of the dried product into pharmaceutical and cosmetic disperse systems, such as gel-type formulations. Thus, developing dried liposomes loaded with vegetable oils or other phytoactive ingredients can promote the development of new high-added-value natural products leveraging Brazilian plant biodiversity.

For cosmetic applications, the constituents of the formulation impact its efficacy. This composition directly influences the product’s physico-mechanical properties, affecting the interaction between the skin and the formulation. Incorporating vegetable oils or other components can alter the texture and rheological properties of the resulting formulations, thereby impacting the skin surface characteristics [16,17].

Modern techniques, such as texture analysis and rheological evaluation, can assess the role of individual ingredients in cosmetic formulations. Conversely, biophysical techniques and skin imaging analysis are valuable, non-invasive, in vivo tools to explore these effects and interactions on the skin. These methodologies offer insights into how cosmetics interact with the skin through various physical principles [16,17,18].

Using a diverse range of methodologies allows for correlating physicochemical parameters with the structure of formulations and their interaction with the skin, including hydration, transepidermal water loss, brightness, and microrelief, which are essential for determining the product’s commercial viability.

In summary, encapsulating the oil of the pequi pulp and other oils from Brazilian plant biodiversity into liposomes and dried liposomes represents an innovative approach for developing novel, effective, and safe raw materials with high added value. This strategy promotes scientific and economic advancement while improving innovation and competitiveness.

This work aims to encapsulate pequi oil in liposomes, evaluate freeze drying for producing dried liposomes, and integrate them into gel-type formulations. Furthermore, the study involves analyzing the rheological behavior and texture profile and assessing the clinical efficacy of these formulations through biophysical techniques and image analysis. The ultimate objective is to valorize Brazilian plant biodiversity by creating advanced cosmetic formulations.

## 2. Materials and Methods

### 2.1. Liposome Manufacture and Characterization

Pequi pulp oil was obtained from Amazon Oil (Ananindeua, Pará, Brazil; 1°22′26.0″ S and 48°23′40.9″ W) using a cold extraction method. The oil is free from preservatives and solvent extraction, making it suitable for use in cosmetics. The substance has a yellow to orange color and its fatty acid composition includes Palmitic Acid, Oleic Acid, Linoleic Acid, and other compounds. The oil has a density of 905.5 ± 0.7 kg/m^3^ at 20 °C and a melting point of 21 °C [3,19,20].

This oil was encapsulated in liposomes to enhance biocompatibility with the skin and facilitate its incorporation into gel-type (aqueous-based) formulations. The formulation’s composition included cholesterol (Sigma-Aldrich, San Luis, AZ, USA), hydrogenated soy phosphatidylcholine (Phospholipon^®^ 90H, Lipoid, Ludwigshafen, Germany), absolute ethyl alcohol, pequi oil (Amazon oil, Pará, Brazil), and Milli-Q^®^ water. Two formulations were developed: liposomes without the active substance (empty—PR1) and liposomes containing 1% (*w*/*w*) pequi oil (PR2), as shown in Table 1.

Liposomal dispersions containing pequi oil were prepared using a method adapted from Jaafar-Maalej et al. (2010) [21] and further refined by our research group [15]. The aqueous phase was heated to 70 °C in a water bath until it reached 60 °C. Concurrently, the oily phase was heated until the lipids melted. Subsequently, the aqueous phase was slowly poured into the oily phase and stirred using an Ultra-Turrax^®^ (IKA Werke, T18 basic, Staufen, Germany) at 22,000 rpm for 5 min.

The formulations were submitted to ultrasonication for particle size reduction using a VibraCell VCX 740 (Sonics & Materials, Newtown, CT, USA) equipped with a 13 mm probe at 45% amplitude. An optimal ultrasonic cycle (5 min on, followed by 2 min off) was determined through a screening assay with PR2 pre-formulation, aiming for the smallest particle size and polydispersity index (PdI). During ultrasonication, samples were maintained in an ice water bath to prevent overheating.

The prepared liposomes were stored at room temperature (25 °C) and subjected to thermal stress tests at 5 °C and 37 °C for stability evaluation. Characterization was performed after 24 h, and on days 7, 14, 28, and 49, to measure parameters such as hydrogenionic potential (pH), color, particle size, polydispersity index (PdI), zeta potential, and encapsulation efficiency (EE), using the methodologies described as follows:

Determination of hydrogenionic potential (pH): The pH of the liposomes was measured in triplicate using a calibrated pH meter (Metrohm, Herisau, Switzerland) with reference pH 4 and pH 7 buffer solutions. The results are reported as mean ± standard deviation.

Color analysis: Sample color was assessed with a Color-Quest XE color spectrophotometer (Hunter Lab, Reston, VA, USA), following the method outlined in Tonon (2009) [22]. Colorimetric analysis was conducted according to CIE coordinates, where the L* parameter indicates brightness, a* denotes the red/green coordinate (with positive values indicating redder shades and negative values indicating greener shades), and b* represents the yellow/blue coordinate (with positive values indicating yellowish tones and negative values indicating bluish tones) [23]. Measurements were taken in quintuplicate.

Particle size, polydispersity index (PdI), and zeta potential: The formulations were diluted at a ratio of 1:1000 (*w*/*w*) with Milli-Q^®^ water and stirred with a magnetic stirrer for 40 min and used to evaluate particle size, polydispersity index, and zeta potential.

Particle size and PdI measurements were conducted using dynamic light scattering (DLS), which evaluates the velocity of particles undergoing Brownian motion [24,25]. For these measurements, 1.0 mL of the diluted solution was placed into a quartz cuvette and analyzed with the Zetasizer Malvern Nano-ZS90 (Malvern Panalytical, Malvern, UK) in Size mode at 25 °C, with data collected in triplicate.

The zeta potential was determined by microelectrophoresis, which measures the particle’s movement in an electric field, allowing for the determination of nanocarrier (NP) charge [24,25]. For data acquisition, 750 µL of the diluted solution was transferred to the disposable capillary cell and analyzed using the Zetasizer Nano—ZS90 in Zeta Potential mode at 25 °C.

Encapsulation efficiency (EE): The absorption spectra of pequi oil diluted in absolute ethanol were monitored using a UV–vis spectrophotometer (HP model 8453, Hewlett-Packard, Palo Alto, USA) over a wavelength range of 190 to 800 nm. To determine the encapsulation efficiency, 20× *g* of liposomes undertook centrifugation at 7000 rpm (5752 rcf) at 25 °C for 90 min, discarding the supernatant. The precipitate was reconstituted in 10 mL of absolute ethanol and submitted to magnetic stirring for 10 min to promote the disruption of the liposome membranes. Subsequently, the solution was filtered through a 0.45 µm Millipore^®^ filter, and the spectrophotometer was employed for sample analysis [26]. The high carotenoid concentration in pequi oil resulted in a characteristic absorbance profile for carotenoids within the 350–500 nm range, setting 448 nm as the wavelength for encapsulation efficiency determination. Encapsulation efficiency was calculated as per Equation (1).

(1) $$EE\left(\%\right)=\left(\frac{{ABS}_{po}}{{ABS}_{PR2}}\right)\times 100$$

In Equation (1), EE is the encapsulation efficiency (in percentage), ABS_po_ is the absorbance of the pequi pulp oil, and ABS_PR2_ is the absorbance of the pequi pulp recovered from the disrupted liposomes after the centrifugation and filtration.

### 2.2. Freeze-Dried Liposome Production and Characterization

The principle of freeze drying involves removing a solvent from a formulation through sublimation. Initially, the sample is frozen at very low temperatures; subsequently, the solvent is removed by sublimation under reduced pressure, followed by a desorption process to eliminate any residual frozen solvent [27]. The freeze-drying conditions were set based on previous research conducted by the LAPROFAR/USP research group [15,28] using sucrose at 10% *w*/*w* relative to the solid content of the liposomes as the cryoprotectant.

Freeze drying was carried out using a lyophilizer (model SNL108B; Thermo Fisher Scientific, Waltham, MA, USA) equipped with a 1.5 L Micromodule lyophilizer unit (305 × 330 × 432 mm), a stainless-steel condenser, a 1/4 hp compressor with 0.30 kW power, a LyoPump VLP195FD ultra-vacuum pump, freeze drying containers, and independent valves. For the freeze-drying process, samples were weighed and frozen at −20 °C for 24 h in 50 mL Falcon tubes, followed by lyophilization for 48 h.

The moisture content of the dried liposomes was measured using the Karl Fischer titration method (Metrohm, Herisau, Switzerland), and their water activity was assessed with the Aqua Lab 4TEV water activity meter (Decagon Devices, Pullman, WA, USA) [29].

Subsequently, the dried products were reconstituted in purified water to their original concentration, following the method described by Bankole et al. (2020) [15]. The mixture was agitated for 60 min using a magnetic stirrer (IKA Werke mod. RT 15, Staufen, Germany). Finally, the pH, particle size, zeta potential, polydispersity index, and encapsulation efficiency were determined by the methods presented in Section 2.1 and compared with the liposomes’ initial values.

The morphologies of the original liposome and redispersed dried liposomes were evaluated by transmission electronic microscopy (TEM) using the procedure presented below.

Morphology of the liposomes and freeze-dried liposomes: The morphological characteristics of the liposomes (PR1 and PR2), each containing 10% sucrose (PR1 + 10% sucrose and PR2 + 10% sucrose), as well as the redispersed dried liposomes, were investigated using transmission electron microscopy (TEM JEOL JEM 2100, Tokyo, Japan). The dried liposomes were resuspended at their initial solid concentrations. Subsequently, all formulations were diluted to a ratio of 1:100 (liposome–water) and carefully applied onto a copper grid. The excess solution was removed using filter paper, and the samples were dried before staining with 3 μL of aqueous uranyl acetate (2% *w*/*v*) for 3 min. Following the drainage of excess uranyl acetate, the samples were dried, and the staining procedure was repeated. The prepared grids were then subjected to TEM analysis [30]. The Microscope is equipped with a LaB6 filament and can reach an acceleration voltage of up to 200 kV. It offers a maximum resolution of 0.23 nm (point) and 0.14 nm (lattice), with a maximum magnification of ×1,500,000.

The obtained images were analyzed using the Image J software 1.8.0. The images were imported into the program and the format was adjusted to 8 bits. Measurement parameters were defined according to Zhang and Wang (2023) [31] to analyze the size and shape of the nanocarriers. The scale was defined and adjusted by tracing a straight line with the same length as the scale bar on each photo. The threshold value was adjusted to aid in separating nanocarriers from the background based on their brightness and contrast, enabling a more precise analysis of the nanocarrier size distribution.

### 2.3. Development of Cosmetic Formulations and Stability Testing

Hydrogels are semisolid systems defined by their three-dimensional polymeric networks, which are stabilized through cross-linked covalent bonds and other weaker cohesive forces, such as hydrogen and ionic bonds. These systems typically consist of a solvent and a gelling agent. The versatility of hydrogels allows for their combination with various chemical compounds, creating cosmetic formulations with a wide range of skin and hair care applications and benefits [32,33].

Prior to developing the cosmetic dried liposome formulation, the sensory properties and interactions among the carrier raw materials were assessed. This evaluation was conducted through a comprehensive review of relevant literature, supplier reports, material safety data sheets (MSDSs), and information from the *Handbook of Pharmaceutical Excipients* [34].

A gel-type formulation was investigated, comprising one variant with empty dried liposomes as a control (vehicle—L1) and another enriched with 4% dried liposome containing pequi pulp oil (*Caryocar brasiliense* Cambess) (L2). To this end, 4% of each dry product was incorporated into the formulations: one gel with the empty dried liposome (L1) and another gel-type loaded with the dried liposome containing pequi oil (L2). Table 2 describes the composition of these cosmetic formulations. To prepare the hydrogel, the polymer and chelating agent were added to the water and mechanically stirred at 500 rpm. The preservative was then homogenized in glycerin and added to the water phase. Finally, the dried liposomes (PR1 or PR2) were added to the formulation under the same stirring conditions for 30 min to ensure homogeneity.

These formulations were subjected to stability testing over 290 days, during which the propensity for phase separation was evaluated using a centrifuge (3 cycles at 3000 rpm for 30 min), and pH levels were monitored with a microprocessor digital pH meter (model R-TEC-7/2-MP, Tecnal, Piracicaba, Brazil).

The gel-type formulations derived from the dried liposomes were characterized through rheology, texture profile, and spreadability analyses; they were also assessed in a short-term clinical efficacy test to evaluate their immediate effect on human skin.

#### 2.3.1. Rheology, Texture Profile, and Spreadability Analyses

The rheology of the dried liposome gel-type compositions was measured in a cone and plate rheometer (Brookfield DV3T, Brookfield, Stoughton, MA, USA) equipped with a CP-52 spindle coupled to Rheocalc T^®^ software. The formulations were kept at room temperature (25 ± 2.0 °C) and analyzed 24 h after preparation. From the rheograms, the minimum apparent viscosity and hysteresis area were determined [35]; the curves were adjusted according to the Ostwald de Waele model to obtain the flow index and consistency index [36]. All analyzes were performed in triplicate.

Texture and spreadability analysis were conducted using the TA.XTplus texture analyzer (Stable Microsystems, Godalming, UK), coupled with Exponent 3.0.5.0 software. Both tests involved the insertion of an analytical probe into the samples [37]. A 35 mm diameter A/BE probe was used for the texture test to evaluate firmness, consistency, cohesiveness, and viscosity index [35,36]. For the spreadability test, a TTC HDP/SR probe was used to determine the shear work of the formulations [35].

#### 2.3.2. Short-Term Clinical Efficacy—Immediate Effect Evaluation

This study was conducted after approval from the Research Ethics Committee Involving Human Beings of the School of Pharmaceutical Sciences of Ribeirão Preto, University of São Paulo (CAAE: 45620321.2.0000.5403—Protocol CEP/FCFRP n° 4.952.770). Ten participants were recruited for the study through the NEATEC laboratory’s clinical study participant database (Núcleo de Estudos Avançados em Tecnologia de Cosméticos). Inclusion criteria for this study were women aged 19 to 30 years, phototypes II and III, healthy, and with normal or oily skin. Exclusion criteria were smokers, lactating women, scars or pigmented lesions on the face that could influence study measurements, and irritation or sensitivity to any formulation component.

Selected participants had the frontal and malar regions marked in four 20 cm^2^ areas, two on each side, with 40 µL of formulation applied to each area. Formulation L1 was applied to one side of the face, and formulation L2 was applied to the other, randomized. In each region, the formulation was spread by the researcher in charge of the study using a disposable finger cot.

The tests began after 20 min of acclimatization in a temperature-controlled environment (20–22 °C) with relative air humidity (45–55%). All participants remained in the room during the study period. Instrumental measurements were taken before (baseline values—T0) and after 2 h (T2) of formulation application, with the determination of transepidermal water loss (TEWL), stratum corneum water content, sebum content, and skin microrelief.

Transepidermal water loss was evaluated using the Tewameter^®^ TM210 equipment (Courage & Khazaka, Köln, Germany), which measures water evaporation from the skin surface based on Adolf Fick’s diffusion principle from 1885. Values were obtained in g/h/m² [16,35].

The aqueous content of the stratum corneum was determined using the Corneometer^®^ CM 825 equipment (Courage & Khazaka, Köln, Germany). This technique is based on the principle of measuring the electrical capacitance of water. Ten measurements were taken in the study region, and the results were presented in arbitrary units (UA) [35].

Sebum content was measured using the Sebumeter^®^ SM810 equipment (Courage & Khazaka, Köln, Germany). This analysis is based on the principles of photometry. An opaque 64 mm^2^ tape was pressed onto the skin for 30 s; sebum collection makes the tape transparent, where the larger transparent area indicates a higher amount of superficial sebum on the skin. Then, the tape is inserted into the device’s opening, and a photocell measures transparency. The values obtained by light transmission represent the amount of sebum on the skin [38,39].

The Visioscan^®^ VC 20plus equipment (Courage & Khazaka, Köln, Germany) was utilized to assess cutaneous microrelief, employing optical profilometry techniques and image scanning with a video camera. This approach provides qualitative and quantitative information about the skin surface under physiological conditions. The Sesm parameter, associated with the shape and width of the skin furrows and indicative of skin smoothness, was evaluated by this procedure [39].

#### 2.3.3. Statistical Analysis

The experimental data obtained were subjected to statistical analysis using Prism GraphPad 8.4.3 (San Diego, CA, USA) and Origin 9.75 (Northampton, MA, USA) software. The Shapiro–Wilk test was used to assess sample normality. For normal distribution, one-way ANOVA with Tukey’s post-test was applied, and for non-normal distribution, the Kruskal–Wallis test with Dunn’s post-test was applied. *p* < 0.05 was considered significant. The results were presented in graphs and figures with discussion based on the data obtained from the literature.

## 3. Results and Discussion

### 3.1. Liposome Manufacture and Characterization

**Figure 1:**
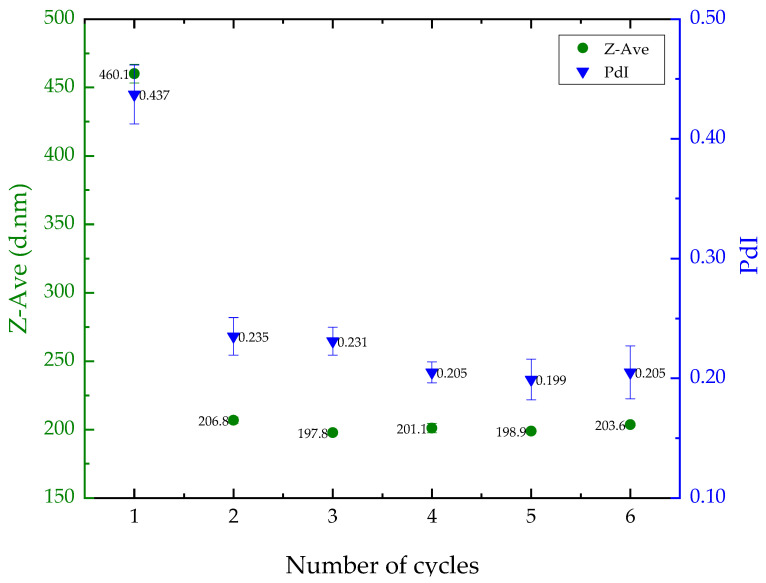
Results for mean liposome particle size (left, in green) and polydispersity index (PdI) (right, in blue) based on the number of sonication cycles.

The impact of ultrasonication cycles on liposome size and polydispersity was assessed using the empty liposome. The results showed a decrease in particle size and PdI after one ultrasonication cycle, but no significant changes (*p* > 0.05) were observed after two cycles (Figure 1). As a result, the liposome preparation protocol was standardized to two cycles, resulting in a Z-Average of approximately 206 nm and a PdI of 0.23. These values are appropriate for delivering nutrients to the skin.

Liposomes may present instabilities over time, such as aggregation, coalescence, and particle fusion, increasing particle size [40]. These formulations are also influenced by temperature, pH, and lipid composition [40]. Therefore, studying the stability of newly developed liposome carriers is relevant, as changes in organoleptic characteristics and pH variations can compromise the viability of formulations, making the product unstable and unsafe.

The liposome pre-formulations developed exhibited low viscosity and pH values in the range of 5 to 6, compatible with skin pH [41]. Colorimetric analysis indicated that all formulations remained stable in brightness and color coordinates (a*: yellow/blue; b*: red/green) throughout the study period and at all temperatures. The gel formulation with PR1 was considered white (L: 88.75 a*: −0.63 b*: −0.99), and the formulation with PR2 was considered yellow (L: 86.62 a*: −0.42 b*: 20.54).

**Figure 2:**
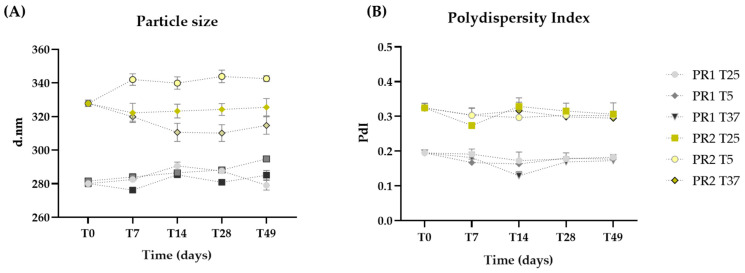
Particle size (**A**) and polydispersity index (**B**) of the empty liposome pre-formulation (PR1) and the liposome pre-formulation with pequi oil (PR2) measured at 24 h (T0), and on days 7 (T7), 14 (T14), 28 (T28), and 49 (T49) after preparation, with storage conditions at room temperature (25 °C—T25), 5 °C (T5), and 37 °C (T37).

The particle sizes of the liposomes are within the nanometric range, with PR1 (284.34 ± 5.38 nm) being smaller than PR2 (326.24 ± 10.25 nm) due to the absence of oil (Figure 2A). Throughout the 49 days, the sizes of formulations PR1 and PR2 remained statistically unchanged (*p* > 0.05), even when subjected to thermal stress.

The literature indicates that particle size is directly related to the penetration of nanoparticles (NPs) into the skin. Typically, healthy skin acts as a barrier, permitting the penetration and permeation of NPs between 4 and 6 nm. NPs smaller than 20 nm can potentially penetrate both healthy and compromised skin—the latter being affected by conditions such as irradiation, inflammation, and infection. In contrast, NPs ranging from 21 to 45 nm are only able to penetrate and permeate damaged skin, while those above 45 nm cannot penetrate or permeate the skin at all [42,43,44,45]. However, cosmetics acting on the surface of the skin can still confer benefits to deeper layers, as external stimuli have an impact on dermal health [46].

The polydispersity index (PdI) quantifies the variance in nanocarrier diameters across a sample’s size distribution, with values ranging from 1 to 0 [47]. A value closer to 0 signifies a higher degree of homogeneity. The incorporation of oil led to increased PdI values, with PR1 and PR2 averaging 0.20 and 0.31, respectively. No significant changes (*p* > 0.05) in PdI were noted throughout the study, even under thermal stress, demonstrating that both pre-formulations preserved their homogeneity (Figure 2B).

Zeta potential (PZ) is a measure of the electric charge on the surface of particles suspended in a solution, where this charge indicates the degree of repulsion between particles, indicating electrokinetic stability [48]. The analysis and monitoring of this parameter are essential, as it can affect the stability and behavior of liposomes in a solution. A higher zeta potential value (in modulus) usually indicates greater repulsion between particles, leading to increased stability of the liposome suspension. On the other hand, a lower zeta potential value can result in liposome aggregation or flocculation [48,49]. In this context, the PZ of both pre-formulations remained stable and close to −30 mV, indicating high electrokinetic stability of the liposomes (Table 3).

The stability testing results showed that both the empty liposome and the liposome containing 1% pequi oil are stable across several parameters, including color, pH, particle size, polydispersity index, and zeta potential.

Spectrophotometric analysis revealed that diluting pequi oil in ethanol at a 1:500 (*v*/*v*) ratio resulted in an absorbance value of 0.16 at a wavelength of 448 nm. Conversely, the liposomes containing pequi oil showed a decrease in absorbance to 0.11 at the same wavelength after their phospholipid membranes were disrupted by the solvent. By comparing the absorbance of the pequi oil with that obtained after the disruption of the liposome membranes, an encapsulation efficiency of 68.8% *w*/*w* was determined by using Equation (1). Therefore, a high amount of pequi oil was successfully encapsulated within the liposomes, highlighting the effectiveness of the encapsulation protocol used here.

### 3.2. Freeze-Dried Liposome Production and Characterization

Various post-processing techniques can enhance liposome stability, bioactivity, and bioavailability, including drying methods such as freeze drying [40]. Freeze drying serves to preserve and stabilize a range of materials. However, it can also lead to alterations in nanocarrier structure and material activity, potentially reducing the effectiveness of the active substances [50]. Consequently, cryoprotectants are often used during freeze drying [40]. Sugars like trehalose and sucrose are popular choices for cryoprotectants because of their protective effectiveness, affordability, non-toxicity, and their ability to enhance the stability and shelf life of freeze-dried products [40,50,51]. Furthermore, these sugars can interact with and replace the water in the polar part of phospholipids, preserving the spacing between these groups during freezing. This action helps reduce the Van Der Waals forces between the acyl chains of phospholipids during freeze drying, thus contributing to liposome stability [40,52].

**Figure 3:**
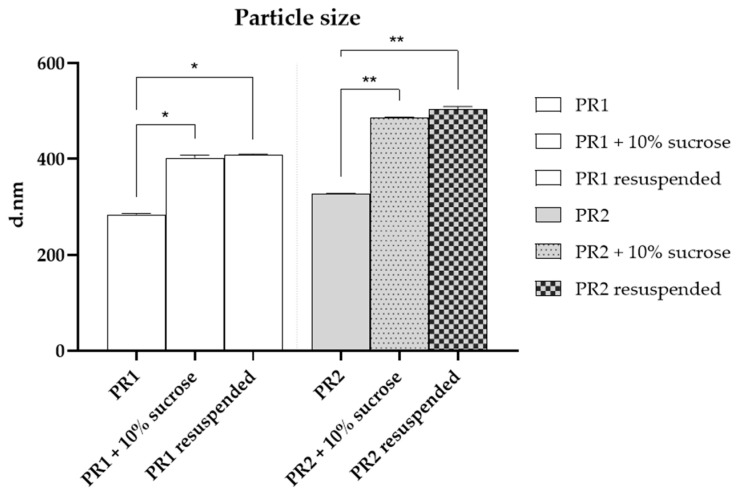
Particle size of the empty liposome—PR1, empty liposome added with 10% of sucrose, resuspended dried liposome, liposome added with 1% of pequi oil—PR2, liposome added with 1% of pequi oil and 10% of sucrose, resuspended dried liposome added with 1% of pequi oil. * Significant difference compared to PR1 (*p* < 0.01); ** Significant difference compared to PR2 (*p* < 0.01).

After the addition of sucrose (10%), a significant increase (*p* < 0.05) in particle size was observed for both pre-formulations, as shown in Figure 3. This increase may be attributed to hydrogen bond interactions between the sugar molecules and the lipid bilayer. The enlargement in particle size upon adding sucrose could suggest the formation of more favorable hydrogen bonds between sucrose and the lipid bilayer or could imply vesicle aggregation. Through molecular dynamics simulations, Roy and co-workers (2016) [53] demonstrated that sucrose molecules can form over 10% more hydrogen bonds with significant phospholipid membrane groups than other sugars, such as trehalose. Consequently, the interaction between sucrose and lipid bilayers engages a greater number of polar heads, enhancing its effectiveness and increasing the liposome particles’ size [53].

Conversely, the resuspended liposomes did not show a significant (*p* > 0.05) variation in particle size compared to those with added cryoprotectants, as illustrated in Figure 3. This observation indicates that the obtained powder can be reconstituted in water while preserving its original particle size.

Regarding the zeta potential (PZ), the presence of sucrose significantly reduced (*p* < 0.05) the zeta potential after freeze-drying, as sugars do not have a charge. Furthermore, sucrose exhibits a lower shielding effect, which may also be correlated with the reduction in the surface charge of nanocarriers [54].

Moisture content and water activity are critical parameters for characterizing and evaluating the stability of dry products. These metrics, dependent on the drying process and the adjuvants used to protect the active compound, are also linked to the product’s hygroscopicity [55]. Moisture content quantifies the total amount of water present in the dry product, with values below 10% indicating a reduced potential for microbial growth and enhanced stability [56]. In this context, the moisture content of PR1 and PR2 after drying remained within the recommended ranges to inhibit microbial growth, with sample PR2 exhibiting a lower value.

Water activity (a_w_) values are associated with the amount of bound and free water in the powder but do not directly indicate its reactivity or mobility [57]. The PR1 powder demonstrated an a_w_ of 0.18, while the PR2 powder had an a_w_ of 0.20. Both values are near the 0.2–0.3 range, within which a lower rate of lipid peroxidation and browning reactions is observed. Values of a_w_ below the activity corresponding to the moisture content of the monolayer are conducive to product stability, as they reduce degradation reactions and microbial growth [58]. Moreover, an a_w_ below 0.3 extends the powder’s shelf life by minimizing biochemical reactions [55]. Therefore, maintaining a low water activity value is imperative for preventing microbial growth, while ensuring it is not excessively low, avoiding the peroxidation of the lipids present in the liposome compositions.

The dried liposomes were redispersed in water at the same initial concentration, and their properties were measured to evaluate their redispersion capability. The results showed no significant (*p* > 0.05) variations in pH, PdI (PR1 + cryoprotectant: 0.25 ± 0.03; PR2 + cryoprotectant: 0.39 ± 0.02), particle size, and zeta potential (PR1 + cryoprotectant: −14.2 ± 1.15 mV; PR2 + cryoprotectant: −15.2 ± 1.67 mV) compared to their original liposomes formulations added with the cryoprotectant. Therefore, the dry product demonstrated resuspension capacity while maintaining the original characteristics of liposomes before freeze drying.

The EE results for the dried liposomes indicate that incorporating 10% sucrose leads to high retention of pequi oil, closely matching the values observed for the original liposome and thus demonstrating minimal loss. The EE was 93.8% compared to the liquid formulation (PR2), highlighting sucrose’s effectiveness in preserving nearly all the initially encapsulated oil.

**Figure 4:**
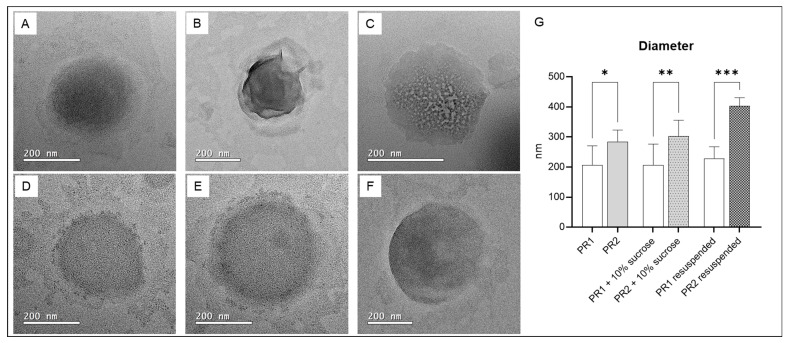
(**A**) Empty liposome—PR1; (**B**) empty liposome added with 10% of sucrose; (**C**) resuspended dried liposome; (**D**) liposome with 1% of pequi oil added—PR2; (**E**) liposome with 1% of pequi oil and 10% of sucrose added; (**F**) resuspended dried liposome with 1% of pequi oil added; (**G**) average diameter of the liposomes. * significant difference compared to PR1 (*p* < 0.01); ** significant difference compared to PR1 + 10% sucrose (*p* < 0.01); *** significant difference compared to PR1 resuspended (*p* < 0.0005).

Figure 4 presents TEM photomicrographs of pure liposomes, liposomes with added cryoprotectant, and resuspended freeze-dried liposomes. The sizes of the pure liposome compositions, as determined by transmission electron microscopy (TEM), are similar to those measured by dynamic light scattering (DLS). However, for liposomes with cryoprotectants and resuspended freeze-dried liposomes, the diameters measured by TEM were smaller than those obtained by DLS. Differences were expected since the size definitions, sample preparation protocols, and measurement methodologies are significantly distinct.

DLS and TEM are two widely employed techniques for nanocarrier characterization, each with unique strengths and limitations. DLS is a fast and non-destructive method for measuring the hydrodynamic size of nanocarriers in solution. It permits the assessment of the size distribution of numerous particles within a sample. In addition, DLS provides valuable information on the Brownian motion and diffusion coefficients of particles. However, its resolution is limited when dealing with particles smaller than 1 nm or larger than 1 µm. In addition, DLS sensitivity to particle shape, polydispersity, and sample preparation conditions can lead to the overestimation of particle size, especially in the presence of aggregates or non-spherical particles [59,60,61].

On the other hand, TEM is a high-resolution imaging technique capable of visualizing individual nanocarriers, providing detailed insight into their morphology, shape, and composition. TEM can achieve atomic resolution and is the preferred choice for detailed morphological analysis. However, TEM requires sample preparation that includes drying and staining, which can alter particle properties [62]. This process is time-consuming and labor-intensive, and TEM is limited to imaging nanocarriers on a solid substrate, not in a solution [61,63].

Both techniques complement each other to provide complete nanocarrier characterization [61].

### 3.3. Rheology, Texture Profile, and Spreadability Analyss of the Gel-Type Formulations

The technological development of cosmetic formulations utilizing pequi oil was registered with the National System for the Management of Genetic Heritage and Associated Traditional Knowledge (SisGen) to ensure compliance with legal and regulatory standards. SisGen (https://sisgen.gov.br/, accessed on 20 January 2022) is a Brazilian system that manages genetic resources and the associated traditional knowledge [64]. A gel-type formulation was developed only after obtaining approval from SisGen (registration number: A15D96E). This registration pertains to using pequi oil derived from the species *Caryocar brasiliense* Cambess. The documentation was completed in compliance with the stipulations of Law 13,123/2015 and its regulatory provisions.

**Figure 5:**
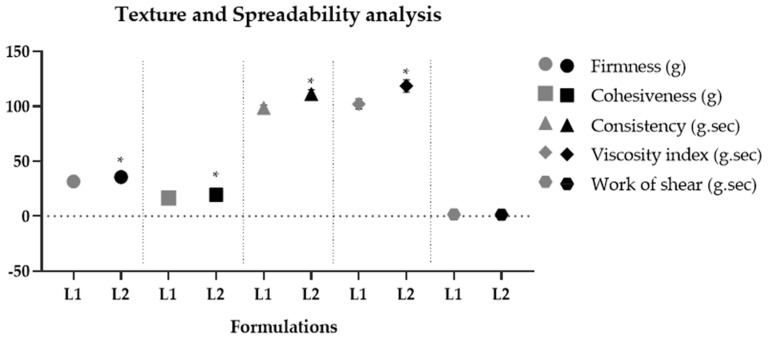
Texture profile − parameters of firmness, cohesiveness, consistency, and shear work − spreadability of vehicle gel (L1) and gel containing 4% dried liposomes with pequi oil (L2) after 24 h of preparation. * significant difference compared to L1 (*p* < 0.05).

Due to the efficient aqueous dispersibility property of dried liposomes, they can be easily incorporated into gel-type cosmetic formulations. The resulting formulations presented an average pH of 5.29 ± 0.08, which is compatible with the skin’s pH [41]. Both formulations maintained their organoleptic characteristics and pH values for 290 days, demonstrating their stability. The rheological behavior of the formulations exhibited a flow index lower than 1 (non-Newtonian), with thixotropy, indicating a recovery of viscosity after shear reduction [35,36]. The hysteresis area results from the thixotropic phenomenon. It indicates qualitatively how long a formulation recovers its viscosity and structure after being subjected to progressively increasing and decreasing shear rates [65]. L1 showed a significantly (*p* < 0.05) smaller hysteresis area compared to L2, indicating that less time is required for these formulations to reorganize when applied on the skin surface [35]. The consistency index and minimum apparent viscosity of L2 were significantly (*p* < 0.05) higher compared to L1, showing that the addition of liposomes containing pequi oil made the formulations more consistent and viscous compared to their vehicle, indicating that the oil phase alters the microstructure of the formulation. Similar results were found in the texture profile analysis, where L2 significantly increased (*p* < 0.05) parameters of firmness, consistency, cohesiveness, and viscosity index (Figure 5). Pequi oil is composed mainly of saturated (40.04% *w*/*w*) and monounsaturated (53.56% *w*/*w*) fatty acids, totaling 93.6% *w*/*w*. Among them, palmitic acid (C16:0) and oleic acid (C18:1) stand out, representing 90.39% *w*/*w* of this total [2]. Decreased unsaturation in the chain promotes lower lipid fluidity and, consequently, a higher melting point [66]. Thus, the presence of these fatty acids in their composition increases the viscosity of the formulation, causing an increase in parameters related to firmness, consistency, and cohesiveness of the gel containing dried liposomes with pequi oil. The rheology parameters can be correlated with the texture profile and spreadability tests. The previous literature has shown that the consistency index and minimum apparent viscosity parameters can positively correlate with the consistency and firmness parameters obtained in the texture test [36]. Thus, both analyzes furnished consistent results, demonstrating the influence of the encapsulated pequi oil on the physico-mechanical properties of cosmetic formulations. The work of shear obtained in the spreadability test showed no significant difference (*p* > 0.05) between the formulations. This parameter is inversely correlated with the spreadability parameter obtained in the sensory properties test [37]. Thus, it is possible to predict possible perceptions of the characteristics of the formulations through instrumental measures. Therefore, no significant (*p* > 0.05) differences were obtained in the work regarding the shear parameter, indicating that the presence of dried liposomes with pequi oil did not compromise the spreadability of the formulation.

### 3.4. Short-Term Clinical Efficacy—Gel-Type Formulations’ Immediate Effect Evaluation

**Figure 6:**
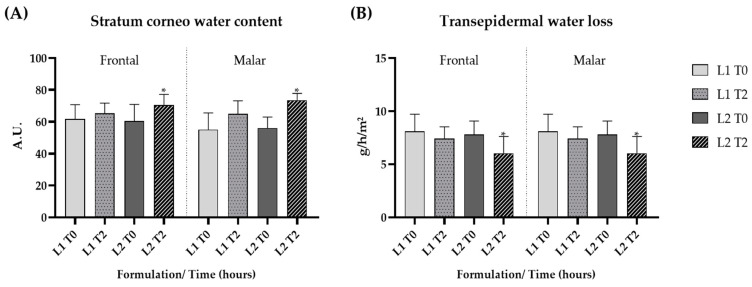
Stratum corneum water content (**A**) and transepidermal water loss (**B**) at the initial time (T0) and 2 h (T2) after the application of the gel formulation (L1) and gel containing dried liposomes with pequi oil (L2). * significant difference compared to baseline values (*p* < 0.05).

The short-term clinical efficacy study showed a significant increase in the stratum corneum water content after the application of formulation L2 (*p* < 0.05), indicating that the addition of the dried liposome loaded with the pequi oil caused a pronounced increase in skin hydration (Figure 6A).

The technique assessing transepidermal water loss (TEWL) is also applied to evaluate the integrity of the skin barrier. TEWL, when combined with measuring the stratum corneum water content, is a reliable indicator of skin hydration and barrier integrity [67]. There was a significant (*p* < 0.05) reduction in TEWL after 2 h of application of formulation L2, which contained pequi oil when compared to baseline values, suggesting that the presence of pequi oil in liposomes improved the skin barrier function (Figure 6B).

Regarding sebum content, no significant alteration (*p* > 0.05) was observed after 2 h of application for both formulations, indicating that adding oil does not affect cutaneous oiliness.

**Figure 7:**
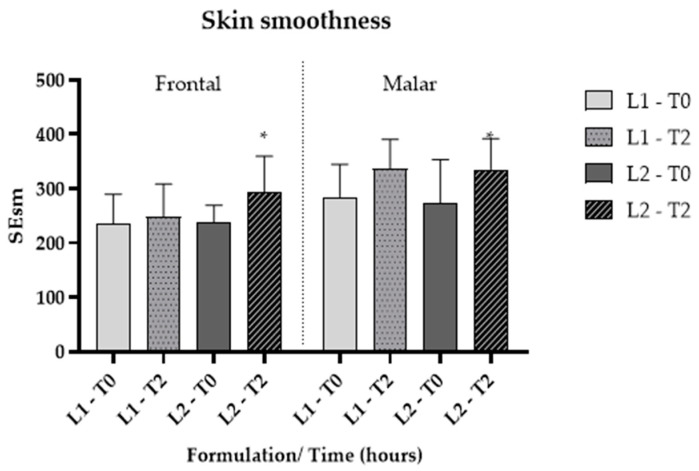
Cutaneous microrelief—Sesm parameter, at the initial time (T0) and 2 h (T2) after application of vehicle gel formulation (L1) and gel containing dried liposomes with pequi oil (L2). * significant difference compared to baseline values (*p* < 0.05).

**Figure 8:**
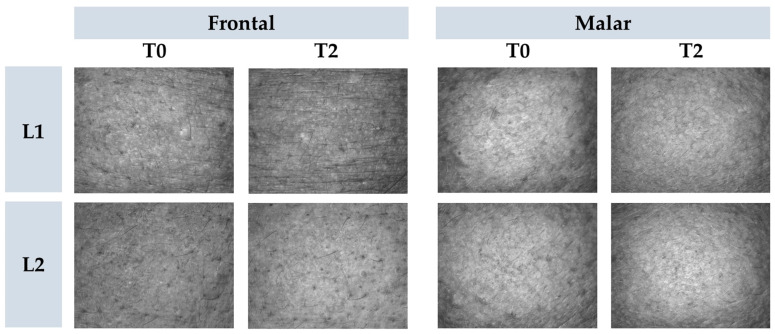
Representative images of cutaneous microrelief at the initial time (T0) and 2 h (T2) after application of vehicle gel formulation (L1) and gel containing dried liposomes with pequi oil (L2).

Skin microrelief was assessed using parameters derived from images captured with Visioscan^®^ FW software. The Sesm parameter quantifies the relationship between the width and depth of wrinkles, where higher values signify improved skin texture and softness [35]. Additionally, skin softness is directly associated with enhanced hydration. The findings indicated that, for L2, there was a significant improvement (*p* < 0.05) in softness compared to L1 (Figure 7 and Figure 8), corroborating the previously mentioned data.

The proposed liposome in this study is composed of soy phosphatidylcholine and cholesterol. Cholesterol is employed to enhance stability, prolonging the entrapment of the active substance within the vesicles [68,69] and increasing the fluidity of the liposome [70]. On the other hand, phosphatidylcholine contributes to the skin’s smoothness [71] and moisturizing effects [72]. While the literature suggests that the inclusion of liposomes, even empty, in cosmetic formulations can lead to improved skin hydration, this study did not observe a significant increase (*p* > 0.05) in the stratum corneum water content.

It is also essential to consider the role of pequi oil in the formulation. Specifically, the fatty acid (FA) profile of vegetable oils is relevant, as it influences the stability, sensory experience, and effects on the skin [73]. Pequi oil, for instance, contains 52.61% oleic acid in its lipid composition. This FA is a natural antioxidant, mitigating oxidative stress, promoting cellular homeostasis, and providing nuclear protection [4,5].

Moreover, vegetable oils, which are water-insoluble emollients, are widely utilized for their potential to soften the skin and impart a pleasing texture. Oils employed in the cosmetic industry often comprise lipids similar to the intercellular lipids found in the skin. Consequently, they can replenish the lipid matrix, repair damaged lipid bilayers, and improve the sensory attributes of formulations [74].

Therefore, encapsulating vegetable oil in liposomes is a prominent strategy for developing innovative cosmetic formulations with high added value. This strategy facilitates the incorporation of lipophilic substances into exclusively aqueous formulations, offering clinical advantages at reduced oil concentrations.

## 4. Conclusions

The freeze-dried liposomes exhibit high dispersibility in water and can be seamlessly incorporated into hydrogel formulations. These formulations demonstrate non-Newtonian pseudoplastic behavior. Instrumental measurements from texture tests revealed that the fatty acid profile of pequi oil enhances the firmness and consistency of the formulation, acting as an oil phase thickener. The clinical efficacy study suggests that the proposed formulation, containing freeze-dried liposomes loaded with pequi pulp oil, is a stable and effective cosmetic product of natural origin with high added value and has the potential to contribute to the preservation of Brazilian biodiversity.

In this context, using phytoactive ingredients in a dry powdered form in cosmetic products presents several benefits, including easy standardization of active ingredient concentration, which enhances reliability and reproducibility. Furthermore, the drying facilitates the production of cosmetic ingredients that can be easily integrated into various cosmetic bases. Highlighting these advantages, we aim to emphasize our findings and their practical implications for cosmetic formulations. Ultimately, this approach has the potential to drive scientific and economic development, promote innovation, enhance competitiveness, help protect Brazilian plant biodiversity, and provide income for traditional communities.

## Figures and Tables

**Table 1:** Composition (%) of the pre-formulations PR1 e PR2.

Phase	Raw Material	Concentration (%)	
		PR1	PR2
Oily	Soy Phosphatidylcholine	5	5
	Cholesterol	1	1
	Absolute Ethyl Alcohol	12	12
	Pequi Pulp Oil (*Caryocar brasiliense* Cambess.)	-	1
Aqueous	Milli-Q^®^ Water	q.s.p.100	q.s.p.100

**Table 2:** Composition of the gel-type formulations (% *w*/*w*).

INCI Name *	Concentration %	
	L1	L2
Milli-Q^®^ water	q.s.p.100	q.s.p.100
Ammonium Acryloyldimethyltaurate/VP Copolymer	0.50	0.50
Glycerin	2.00	2.00
Disodium EDTA	0.05	0.05
Xylityl Sesquicaprylate	0.80	0.80
Freeze-dried liposome PR1 (empty)	4.00	-
Freeze-dried liposome PR2 (1% Pequi oil—*Caryocar brasiliense* Cambess pulp oil)	-	4.00

* International nomenclature of cosmetic ingredients.

**Table 3:** Zeta potential results (mV) of the developed formulations at different storage periods and temperatures.

Time (Days)	Liposome Pre-Formulations/Storage Temperature (°C)					
	PR1 T5	PR1 T25	PR1 T37	PR2 T5	PR2 T25	PR2 T37
T0	−28.43	−28.43	−28.43	−29.87	−29.87	−29.87
T7	−35.10	−31.13	−31.47	−33.03	−34.40	−33.73
T14	−34.07	−31.00	−29.60	−30.63	−33.60	−30.37
T28	−31.10	−33.47	−29.60	−30.13	−36.73	−30.20
T49	−34.33	−31.03	−29.20	−31.93	−30.07	−30.60

## Data Availability

Please contact the authors directly for access to the data.

## References

[1] Ferreira P.M.P., Arcanjo D.D.R., Peron A.P. (2023). Drug development, Brazilian biodiversity and political choices: Where are we heading?. J. Toxicol. Environ. Part B.

[2] Silva G.T., Di Pietro Fernandes C., Hiane P.A., Freitas K.D.C., Figueiredo P.S., Inada A.C., Filiú W.F., Maldonade I.R., Nunes A.A., de Oliveira L.C.S. (2020). Caryocar brasiliense Cambess. Pulp Oil Supplementation Reduces Total Cholesterol, LDL-c, and Non-HDL-c in Animals. Molecules.

[3] Pegorin G.S.A., Marques M.O.M., Mayer C.R.M., Santos L. (2020). Development of a Phytocosmetic Enriched with Pequi (*Caryocar brasiliense* Cambess) Oil. Braz. Arch. Biol. Technol..

[4] De Figueiredo P.R.L., Oliveira I.B., Neto J.B.S., de Oliveira J.A., Ribeiro L.B., de Barros Viana G.S., Rocha T.M., Leal L.K.A.M., Felipe C.F.B., Coutinho H.D.M. (2016). *Caryocar coriaceum* Wittm. (Pequi) fixed oil presents hypolipemic and anti-inflammatory effects in vivo and in vitro. J. Ethnopharmacol..

[5] Pires J., Cargnin S.T., Costa S.A., Sinhorin V.D.G., Damazo A.S., Sinhorin A.P., de Campos Bicudo R., Cavalheiro L., de Souza Valladão D.M., Pohlmann A.R. (2020). Healing of dermal wounds property of *Caryocar brasiliense* oil loaded polymeric lipid-core nanocapsules: Formulation and in vivo evaluation. Eur. J. Pharm. Sci..

[6] Nascimento-Silva N.R.R.D., Naves M.M.V. (2019). Potential of Whole Pequi (*Caryocar* spp.) Fruit—Pulp, Almond, Oil, and Shell—As a Medicinal Food. J. Med. Food.

[7] Machado M., Rodriguez-Alcalá L.M., Gomes A.M., Pintado M. (2022). Vegetable oils oxidation: Mechanisms, consequences and protective strategies. Food Rev. Int..

[8] Sherry M., Charcosset C., Fessi H., Greige-Gerges H. (2013). Essential oils encapsulated in liposomes: A review. J. Liposome Res..

[9] Yang S., Liu L., Han J., Tang Y. (2020). Encapsulating plant ingredients for dermocosmetic application: An updated review of delivery systems and characterization techniques. Int. J. Cosmet. Sci..

[10] Silva G.S., Jange C.G., Rocha J.S., Chaves M.A., Pinho S.C. (2017). Characterisation of curcumin-loaded proliposomes produced by coating of micronised sucrose and hydration of phospholipid powders to obtain multilamellar liposomes. Int. J. Food Sci..

[11] Lammarı N., Louaer O., Meniai A.H., Fessi H., Elaıssarı A. (2021). Plant oils: From chemical composition to encapsulated form use. Int. J. Pharm..

[12] Figueroa-Robles A., Antunes-Ricardo M., Guajardo-Flores D. (2021). Encapsulation of phenolic compounds with liposomal improvement in the cosmetic industry. Int. J. Pharm..

[13] Manca M.L., Matricardi P., Cencetti C., Peris J.E., Melis V., Carbone C., Escribano E., Zaru M., Fadda A.M., Manconi M. (2016). Combination of argan oil and phospholipids for the development of an effective liposome-like formulation able to improve skin hydration and allantoin dermal delivery. Int. J. Pharm..

[14] Chen Y., Yi X., Pan M.H., Chiou Y.S., Li Z., Wei S., Yin X., Ding B. (2021). The interaction mechanism between liposome and whey protein: Effect of liposomal vesicles concentration. J. Food Sci..

[15] Bankole V.O., Osungunna M.O., Souza C.R.F., Salvador S.L., Oliveira W.P. (2020). Spray-Dried Proliposomes: An innovative method for encapsulation of *Rosmarinus officinalis* L. polyphenols. AAPS PharmSciTech.

[16] Melo M.O., Maia Campos P.M.B.G. (2019). Application of biophysical and skin imaging techniques to evaluate the film-forming effect of cosmetic formulations. Int. J. Cosmet. Sci..

[17] Faucheux E., Picard C., Grisel M., Savary G. (2020). Residual film formation after emulsion application: Understanding the role and fate of excipients on skin surface. Int. J. Pharm..

[18] Eudier F., Savary G., Grisel M., Picard C. (2019). Skin surface physico-chemistry: Characteristics, methods of measurement, influencing factors and future developments. Adv. Colloid Interface Sci..

[19] Silva T.A., de Assunção R.M.N., Vieira A.T., de Oliveira M.F., Batista A.C.F. (2014). Methylic and ethylic biodiesels from pequi oil (*Caryocar brasiliense* Camb.): Production and thermogravimetric studies. Fuel.

[20] Amazon Oil Specification of Virgin Pequi Oil (2021). https://amazonoil.com.br/produtos-da-floresta/pequi-cariocar-brasiliensis/.

[21] Jaafar-Maalej C., Diab R., Andrieu V., Elaissari A., Fessi H. (2010). Ethanol injection method for hydrophilic and lipophilic drug-loaded liposome preparation. J. Liposome Res..

[22] Tonon R.V. (2009). Secagem por Atomização do Suco de Açai: Influência das Variáveis de Processo, Qualidade e Estabilidade do Produto. Doctoral Thesis.

[23] Šeremet D., Štefančić M., Petrović P., Kuzmić S., Doroci S., Mandura Jarić A., Cebin A.V., Pjanović R., Komes D., Komes D. (2022). Development, Characterization and Incorporation of Alginate-Plant Protein Covered Liposomes Containing Ground Ivy (*Glechoma hederacea* L.) Extract into Candies. Foods.

[24] Bhattacharjee S. (2016). DLS and zeta potential–what they are and what they are not?. J. Control. Release.

[25] Baldim I., Tonani L., Von Zeska Kress M.R., Oliveira W.P. (2019). *Lippia sidoides* essential oil encapsulated in lipid nanosystem as an anti-Candida agent. Ind. Crops Prod..

[26] Meinhardt-Wollweber M., Suhr C., Kniggendorf A.-K., Roth B. (2018). Absorption and resonance Raman characteristics of β-carotene in water-ethanol mixtures, emulsion and hydrogel. AIP Adv..

[27] Assegehegn G., Brito-de la Fuente E., Franco J.M., Gallegos C. (2019). The importance of understanding the freezing step and its impact on freeze-drying process performance. J. Pharm. Sci..

[28] Baldim I., Oliveira A.M., Souto E.B., Oliveira W.P. (2022). Cyclodextrins-in-liposomes: A promising delivery system for *Lippia sidoides* and *Syzygium aromaticum* essential oils. Life.

[29] Secolin V.A., Souza C.R.F., Oliveira W.P. (2017). Spray drying of lipid-based systems loaded with *Camellia sinensis* polyphenols. J. Liposome Res..

[30] Dos Santos A.M., Meneguin A.B., Fonseca-Santos B., de Souza M.P.C., Ferreira L.M.B., Sabio R.M., Chorilli M., Gremião M.P.D. (2020). The role of stabilizers and mechanical processes on physico-chemical and anti-inflammatory properties of methotrexate nanosuspensions. J. Drug Deliv. Sci. Technol..

[31] Zhang S., Wang C. (2023). Precise analysis of nanoparticle size distribution in TEM image. Methods Protoc..

[32] Mitura S., Sionkowska A., Jaiswal A. (2020). Biopolymers for hydrogels in cosmetics: Review. J. Mater. Sci. Mater. Med..

[33] Kim B., Cho H.E., Moon S.H., Ahn H.-J., Bae S., Cho H.-D., An S. (2020). Transdermal delivery systems in cosmetics. Biomed. Dermatol..

[34] Sheskey P.J., Hancock B.C., Moss G.P., Goldfarb D.J. (2020). Handbook of Pharmaceutical Excipients.

[35] Kakuda L., Campos P.M.B.G., Zanin R.B., Favaro L.N. (2023). Development of multifunctional sunscreens: Evaluation of physico-mechanical and film-forming properties. Int. J. Pharm..

[36] Calixto L.S., Maia Campos P.M.B.G. (2017). Physical-Mechanical characterization of cosmetic formulations and correlation between instrumental measurements and sensorial properties. Int. J. Cosmet. Sci..

[37] Calixto L.S., Infante V.H.P., Maia Campos P.M.B.G. (2018). Design and characterization of topical formulations: Correlations between instrumental and sensorial measurements. AAPS PharmSciTech.

[38] Leite M.G.A., Campos P.M. (2020). Correlations between sebaceous glands activity and porphyrins in the oily skin and hair and immediate effects of dermocosmetic formulations. J. Cosm. Dermatol..

[39] Shirata M.M.F., Maia Campos P.M.B.G. (2017). Influence of UV filters on the texture profile and efficacy of a cosmetic formulation. Int. J. Cosmet. Sci..

[40] Yu J.Y., Chuesiang P., Shin G.H., Park H.J. (2021). Post-processing techniques for the improvement of liposome stability. Pharmaceutics.

[41] Infante V.H.P., Leite M.G.A., Maia Campos P.M. (2022). Film-Forming Properties of Topical Formulations for Skin and Hair: In Vivo and In Vitro Studies Using Biophysical and Imaging Techniques. AAPS PharmSciTech.

[42] Liu J., Zheng A., Peng B., Xu Y., Zhang N. (2021). Size-dependent absorption through stratum corneum by drug-loaded liposomes. Pharm. Res..

[43] Try C., Moulari B., Béduneau A., Fantini O., Pin D., Pellequer Y., Lamprecht A. (2016). Size dependent skin penetration of nanoparticles in murine and porcine dermatitis models. Eur. J. Pharm. Biopharm..

[44] Filon F.L., Mauro M., Adami G., Bovenzi M., Crosera M. (2015). Nanoparticles skin absorption: New aspects for a safety profile evaluation. Regul. Toxicol. Pharmacol..

[45] Baroli B. (2010). Penetration of nanoparticles and nanomaterials in the skin: Fiction or reality?. J. Pharm. Sci..

[46] Yoshida M., Shin K.O., Muraoka S., Choi Y., Park J.H., Park S.-H., Hwang J.-T., Park K., Uchida Y. (2023). The Epidermal Environment’s Influence on the Dermal Environment in Response to External Stress. Skin Pharmacol. Physiol..

[47] Mattos M.V.C.V., Michelon M., Burkert J.F.M. (2022). Production and stability of food-grade liposomes containing microbial carotenoids from *Rhodotorula mucilaginosa*. Food Struct..

[48] Agustinisari I., Mulia K., Nasikin M. (2020). The effect of eugenol and chitosan concentration on the encapsulation of eugenol using whey protein–maltodextrin conjugates. Appl. Sci..

[49] Xu Y., Wei Y., Jiang S., Xu F., Wang H., Shao X. (2022). Preparation and characterization of tea tree oil solid liposomes to control brown rot and improve quality in peach fruit. LWT.

[50] Boafo G.F., Magar K.T., Ekpo M.D., Qian W., Tan S., Chen C. (2022). The Role of Cryoprotective Agents in Liposome Stabilization and Preservation. Int. J. Mol. Sci..

[51] Jangle R.D., Thorat B.N. (2013). Effect of freeze-thawing study on curcumin liposome for obtaining a better freeze-dried product. Dry. Technol..

[52] Marchianò V., Matos M., Serrano E., Álvarez J.R., Marcet I., Blanco-López M.C., Gutiérrez G. (2022). Lyophilised nanovesicles loaded with vitamin B12. J. Mol. Liq..

[53] Roy A., Dutta R., Kundu N., Banik D., Sarkar N. (2016). A comparative study of the influence of sugars sucrose, Trehalose, and maltose on the hydration and diffusion of DMPC lipid bilayer at complete hydration: Investigation of structural and spectroscopic aspect of lipid–sugar interaction. Langmuir.

[54] Varshosaz J., Eskandari S., Tabbakhian M. (2012). Freeze-drying of nanostructure lipid carriers by different carbohydrate polymers used as cryoprotectants. Carbohydr. Polym..

[55] Misra S., Pandey P., Dalbhagat C.G., Mishra H.N. (2022). Emerging technologies and coating materials for improved probiotication in food products: A review. Food Bioprocess Technol..

[56] Chaves M.A., Pinho S.C. (2020). Unpurified soybean lecithins impact on the chemistry of proliposomes and liposome dispersions encapsulating vitamin D3. Food Biosci..

[57] Rahman M.S. (2009). Food stability beyond water activity and glass transition: Macro-micro region concept in the state diagram. Int. J. Food Prop..

[58] Labuza T.P., Altunakar B. (2020). Water activity prediction and moisture sorption isotherms. Water Act. Foods Fundam. Appl..

[59] Hinterwirth H., Wiedmer S.K., Moilanen M., Lehner A., Allmaier G., Waitz T., Lindner W., Lämmerhofer M. (2013). Comparative method evaluation for size and size-distribution analysis of gold nanoparticles: Other Techniques. J. Sep. Sci..

[60] Yeap S.P., Lim J., Ngang H.P., Ooi B.S., Ahmad A.L. (2018). Role of Particle–Particle Interaction Towards Effective Interpretation of Z -Average and Particle Size Distributions from Dynamic Light Scattering (DLS) Analysis. J. Nanosci. Nanotechnol..

[61] Filippov S.K., Khusnutdinov R., Murmiliuk A., Inam W., Zakharova L.Y., Zhang H., Khutoryanskiy V.V. (2023). Dynamic light scattering and transmission electron microscopy in drug delivery: A roadmap for correct characterization of nanoparticles and interpretation of results. Mater. Horiz..

[62] Shah R., Eldridge D., Palombo E., Harding I. (2015). Lipid Nanoparticles: Production, Characterization and Stability.

[63] Ito T., Sun L., Bevan M.A., Crooks R.M. (2004). Comparison of nanoparticle size and electrophoretic mobility measurements using a carbon-nanotube-based coulter counter, dynamic light scattering, transmission electron microscopy, and phase analysis light scattering. Langmuir.

[64] Mozini L.M., Chege Kamau E. (2022). Brazilian Biodiversity Law: Challenges and Opportunities for Industries and Research Institutions. Ius Gentium: Comparative Perspectives on Law and Justice.

[65] César F.C., Maia Campos P.M. (2020). Influence of vegetable oils in the rheology, texture profile and sensory properties of cosmetic formulations based on organogel. Int. J. Cosmet. Sci..

[66] Onyeike E.N., Acheru G.N. (2002). Chemical composition of selected Nigerian oil seeds and physicochemical properties of the oil extracts. Food Chem..

[67] Kakuda L., Maia Campos P.M.B.G. (2023). Aplicação do Óleo de Pequi para Cuidados da Pele. Cosmet. Toilet..

[68] Ashtiani H.R.A., Bishe P., Lashgari N.A., Nilforoushzadeh M.A., Zare S. (2016). Liposomes in cosmetics. J. Skin Stem Cell.

[69] Sułkowski W.W., Pentak D., Nowak K., Sułkowska A. (2005). The influence of temperature, cholesterol content and pH on liposome stability. J. Mol. Struct..

[70] Srihera N., Li Y., Zhang T.T., Wang Y.M., Yanagita T., Waiprib Y., Xue C.H. (2022). Preparation and characterization of astaxanthin-loaded liposomes stabilized by sea cucumber sulfated sterols instead of cholesterol. J. Oleo Sci..

[71] Bilal M., Iqbal H.M. (2020). New insights on unique features and role of nanostructured materials in cosmetics. Cosmetics.

[72] Dubey S.K., Dey A., Singhvi G., Pandey M.M., Singh V., Kesharwani P. (2022). Emerging trends of nanotechnology in advanced cosmetics. Colloids Surf. B Biointerfaces.

[73] Lodén M. (2003). Role of topical emollients and moisturizers in the treatment of dry skin barrier disorders. Am. J. Clin. Dermatol..

[74] Mawazi S.M., Ann J., Othman N., Khan J., Alolayan S.O., Al Thagfan S.S., Kaleemullah M. (2022). A Review of Moisturizers; History, Preparation, Characterization and Applications. Cosmetics.

